# Cryptic genomic lesions in adverse-risk acute myeloid leukemia identified by integrated whole genome and transcriptome sequencing

**DOI:** 10.1038/s41375-019-0546-1

**Published:** 2019-08-21

**Authors:** Jaeseung C. Kim, Philip C. Zuzarte, Tracy Murphy, Michelle Chan-Seng-Yue, Andrew M. K. Brown, Paul M. Krzyzanowski, Adam C. Smith, Faiyaz Notta, Mark D. Minden, John D. McPherson

**Affiliations:** 1 Department of Medical Biophysics, Faculty of Medicine, University of Toronto, Toronto, ON Canada; 2 Genomics Technology Program, Ontario Institute for Cancer Research, Toronto, ON Canada; 3 Princess Margaret Cancer Centre, University Health Network, Toronto, ON Canada; 4 PanCuRx Translational Research Initiative, Ontario Institute for Cancer Research, Toronto, ON Canada; 5 Department of Laboratory Medicine and Pathobiology, University of Toronto and the Laboratory Medicine Program, University Health Network, Toronto, ON Canada; 6 Biochemistry and Molecular Medicine, University of California, Davis, Sacramento, CA USA

**Keywords:** Acute myeloid leukaemia, Cancer genomics, Cytogenetics

## To the Editor:

Acute myeloid leukemia (AML) is a heterogeneous group of hematologic malignancies characterized by the proliferation of myeloid cells blocked in their ability to differentiate. Evaluation by G-banding and fluorescence in situ hybridization is an essential aspect in the initial disease characterization and even now is fundamental in identifying cytogenetic abnormalities that can inform disease diagnosis, prognosis, and treatment decision [1, 2]. For instance, the identification of t(8;21)(q22;q22) or t(15;17)(q22;q12), which generate *RUNX1-RUNX1T1* or *PML-RARA* gene fusions, respectively, confers favorable prognosis when treated accordingly [1]. In recent years, advancements in next-generation sequencing and efforts by large genomics studies have led to a classification of 11 AML subgroups based on cytogenetic abnormalities as well as mutations in genes, such as *NPM1* or *CEBPA* [1].

AML with a complex karyotype, defined by the presence of three or more unrelated chromosomal aberrations and the absence of favorable cytogenetic rearrangements, is associated with *TP53* mutations and strikingly poor outcome [1, 3]. While conventional cytogenetics is still a powerful technique, complex chromosomal aberrations test the limits of cytogenetic resolution. Moreover, detection of cryptic genomic lesions, especially gene fusions, by orthogonal methods can lead to adjustment of treatment regimens and/or identification of biomarkers [4]. The frequency at which cryptic gene fusions are present within complex karyotypes is currently unknown [5]. In this study, we investigated whether whole-genome sequencing (WGS) and whole-transcriptome sequencing (RNA-seq) can resolve these complex aberrations in a series of patients where conventional cytogenetics could not resolve the driver event. Sequencing was performed on nine patients with adverse-risk AML; seven cases had complex karyotypes and the others had unusual translocation events (Table 1 and Supplementary Table 1). The study was approved by the Research Ethics Board of the University Health Network (REB# 01–0573) and written informed consent was obtained from all patients.

**Table 1:** Comparison of cytogenetic and genomic findings from nine patients with adverse-risk AML

Case	Karyotype^a^	Complex karyotype	Composite karyotype	No. of cytogenetic abnormalities		CNVs^b^	Chromosomal rearrangements^b^	Gene fusions	SNVs
				Total	NGS concordant				
1	51, XY, **+Y**, **+der(1;7)(q10;p10)**, **+6**, **+8**, **+10** [10]	Yes		5	5	Gains: **1q**, **6**, **7p**, **8**, **10**, **Y**			*RUNX1, PTPN11, BCOR*
2	45, XX, **-7** [17] / 45, **X**, **t(X;8)(p21.2;q24.1)**, **-7** [2] / 46, XX[1]			2	2	Losses: **7**, *BCOR*	**t(X;8)(p11.4;q24.13)** linked to *BCOR* deletion; t(3;15)(p24.3;q14)		*IDH2*
3	43~45, **X**, **-Y**, t(1;5)(q21;p13), t(1;6)(q21;p25), -2, **add(2)(p21)**, **add(3)(q27)**, **del(3)(q27)**, add(4)(p16), del(4)(q33), del(5)(q31), add(6)(p25), del(6)(q21), -7, add(7)(q36), **del(7)(q22)**, -8, **add(8)(q24.3)**, del(8)(?q23), +del(8)(q22), add(9)(p22), add(9)(q34), add(11)(p15), **add(12)(p13)**, -16, add(16)(p13.3), **-17**, **der(17)t(11;17)(q13;p13)**, add(18)(q23), der(19)t(11;19)(q13;q13.3), -21, add(22)(p11.2), +4mar [cp20]	Yes	Yes	31	9	Gains: 1p36.33-p35.1, 2p25.3-p22.3, **2p21-p11.2** (*BCL11A*), **3q26.32-q29**, **8q12.1-q23.1** (*RUNX1T1*), **11q13.4-q25** (*ETS1*), **12p13.33-p13.2**, 17q25.1-q25.3 (*SRSF2*), 19p13.3, 20q13.32-q13.33 Losses: 2q11.2-q31.3, **7q21.3-q36.2**, 11p15.5-p13 (*WT1*), 16q13-q24.3 (*CTCF*), **17p13.3-p12** (*TP53*), **17q11.2-q24.3** (*NF1*), 19q13.42-q13.43, **Yq11.221-q12**	t(1;5)(q42.3;q12.3); t(3;12) (q26.2;p13.2) linked to gene fusion; t(9;20)(q34.11;q13.12); der(Y)t(Y;3)(q11.221;q26.32) linked to 3q gain and Yq loss; chr4/7 rearrangements linked to 7q loss; chr**2**/**3**/**8**/**11**/**12**/16/**17**/ 19/22/X rearrangements linked to CNVs	*ETV6-MECOM*	*TP53*
4	48-49, XX, **+6**, **+8**, **+9**, **t(12;17)(p13;q11.2)**, i(17)(q10), **inv(18)(q11.2q21)** [cp20]	Yes	Yes	6	5	Gains: **6**, **8**, **9** Losses: 17p13.3-p13.1 (*TP53*), **18q21.2** (*SMAD4*)	t(11;**12**;**17**)(p15.4;**p13.3**;**q11.2**) linked to gene fusion (Figure 1)	*NUP98-KDM5A*	*ASXL1*
5	46, XY, **del(6)(q21q23)**, **t(10;11)(p1?2;q21)**, **inv(12)(q15q24.1)**, del(15)(q2?4) [cp19] / 46, XY [1]	Yes	Yes	4	3	Losses: *ARPP21*, *BBX*, **6q16.1-q22.31** (*FOXO3*), *MACC1*, 8p12-p11.21 (*FGFR1*), 8q11.21-q11.23, *SH2B3*, *TBX3*, *MIR5009*, *SETD4*	**t(10;11)**(p12.3;q14.2) linked to gene fusion; t(7;18) linked to *MACC1* del; **chr12 rearrangements** linked to *SH2B3* and *TBX3* deletions	*PICALM-MLLT10*; *FIP1L1-PDGFRA* (only in relapse)	
6	46, XX, **del(5)(q31)**, **t(11;12)(p13;p13)** [4] / 45, **idem**, **-13** [5] / 46, XX [1]	Yes		3	3	Losses: *FHIT*, *CDH18*, **5q22.2q32** (*APC*), *LACE1/FOXO3*, 11p13 (*WT1*), 11p13-p12, 12p13.2-p12.3 (*ETV6*), 12p11.1, 12q12, **13**, *SLC47A1*	chr**11**/**12**/17 rearrangements linked to CNVs and gene fusion (Figure 1)	*NUP98-BPTF*	*WT1, ETV6*
7	43~48, XY, +?Y, +?Y, **der(5)t(5;17)(q1?3;q11.2)**, **-7**, **+8**, **-12**, -13, **t(16;18)(q24;q23)**, +1~2mar [cp10]	Yes	Yes	8	5	Gains: **8**, 13q12.11-q12.2 (*FLT3*) Losses: 1p36.12-p36.11 (*RPL11*), 1p35.3-p35.2, 1p22.1-p21.3, 2p13.3-p13.1, 2p12-p11.2, 2q32.2-q37.3 (*SF3B1*), 5q11.2-q35.3 (*APC*, *NPM1*), **7**, **12p13.2-p12.1** (*ETV6*), **12p12.1-p11.21**, **12q12-q24.13**, *TCF12*, 16q21-q22.2 (*CTCF*), 16q22.3-q24.3, 17p13.3-p11.2 (*TP53*), 17q11.2-q12 (*NF1*), 18p11.31, 18q22.3-q23	chr1 rearrangements linked to three segmental losses; chr2/**5**/8/**12**/13/**16**/**17**/**18** rearrangements linked to other CNVs; inv(7)(q11.22q36.3)		*TP53*
8	42-45, X, der(X)t(X;3)(q28;q21), **der(1)t(1;17)(p35;q21)**, **-3**, -5, **add(8)(q13)**, -9, add(11)(q13), add(12)(q24), **i(13)(q10)**, **del(16)(q23)**, -17, -18, -19, **+idic(22)(p12)**, +2r, +mar1 [cp9] / 46, XX [1]	Yes	Yes	14	6	Gains: 3q13.33-q29, **8q** (*MYC*), **13**, **22** Losses: **3**, 5q31.1, 5q31.2-q35.3 (*NPM1*), *SMARCA2*, 9p13.2-p11.2 (*PAX5*), 9q33.1-q33.3, 12p13.33-p11.23 (*ETV6*), **16q21-q24.3** (*CTCF*), 17p13.2-p11.2 (*TP53*), 17q11.2-q12 (*NF1*), 19p13.2-p13.12 (*JUNB*), *BCOR*, *KDM6A*	chr**1**/5/**8**/9/11/12/**16**/**17**/19 rearrangements linked to CNVs		*TP53*
9	46, XX, **t(1;14)(q21;q11.2)** [20]			1	1		**t(1;14)(q23.2;q12)** linked to truncation of *ATP1A2*		*DNMT3A, FLT3, TET2*

^a^ Bold font in karyotype denotes cytogenetic abnormalities that are exactly or closely matched by the NGS findings, which are enumerated in the “NGS concordant” column.

^b^ Bold font in CNVs and chromosomal rearrangements denotes NGS findings that match or may explain the cytogenetic abnormalities. For CNVs, gains and losses are listed for chromosomes (i.e. 7), chromosome arms (i.e. 7p), subchromosomal segments (i.e. 12p13.2), and genes (i.e. *BCOR*). Key genes for large segments are shown in brackets when applicable.

WGS and RNA-seq libraries were generated using NEBNext DNA Library Prep (New England Biolabs) and TruSeq Stranded Total RNA Sample Prep (Illumina), respectively, and were sequenced on the Illumina HiSeq 2000 platform. Bioinformatic tools used here are listed in Supplementary Table 2. Briefly, we used BWA-MEM for WGS alignment, CREST for structural variant (SV) calling, and HMMcopy for copy number variation (CNV) detection. In parallel, we used STAR for RNA-seq alignment, STAR-Fusion for fusion transcript detection, and GATK for SNV/indel calling from RNA-seq data. By performing low-coverage WGS (mean genome coverage of 11.3×; Supplementary Table 1) of leukemia DNA without matched germline DNA, we aimed to detect SVs and CNVs in a manner comparable to conventional cytogenetics. Furthermore, we compared gene fusions and rearrangements detected by CREST with fusion transcripts detected by STAR-Fusion to cross validate. DNA breakpoints and fusion transcripts were validated by PCR and RT-PCR, respectively, followed by Sanger sequencing.

Analysis of the leukemia genomes concordantly identified 39 out of 74 (53%) cytogenetic abnormalities annotated by visual inspection (Table 1 and Supplementary Table 3). The concordance rates for translocations/inversions, chromosomal gains/losses, and subchromosomal gains/losses were 74%, 54%, and 37%, respectively (Supplementary Table 1). For five cases with composite karyotypes, which were used to capture the karyotypic heterogeneity, their average concordance rate was lower than that of the other four cases (59% vs. 100%; Wilcoxon rank-sum test, *p* = 0.015). This is likely due to the challenge of detecting subclonal CNVs and SVs with low-coverage WGS. Nonetheless, many cryptic genomic lesions, including submicroscopic deletions of *BCOR*, *TP53*, and *FOXO3/LACE1*, that affect known leukemia-causing/modifying genes were detected. For instance, t(X;8)(p21.2;q24.1) that was reported following conventional cytogenetics in case 2 was revealed by genomic investigation to be linked to two other SVs that resulted in the deletion of *BCOR* (Supplementary Fig. 1). Complex and unbalanced rearrangement patterns, which can be more difficult to discern by G-banded karyotyping, were detected in five cases and could be linked to cytogenetic abnormalities resembling the actual events. Furthermore, in accordance with known association between *TP53* mutation and complex karyotype [3], each of the three cases with the most cytogenetic abnormalities—cases 3, 7, and 8—had a point mutation and a copy number loss of *TP53* (Table 1).

**Fig. 1:**
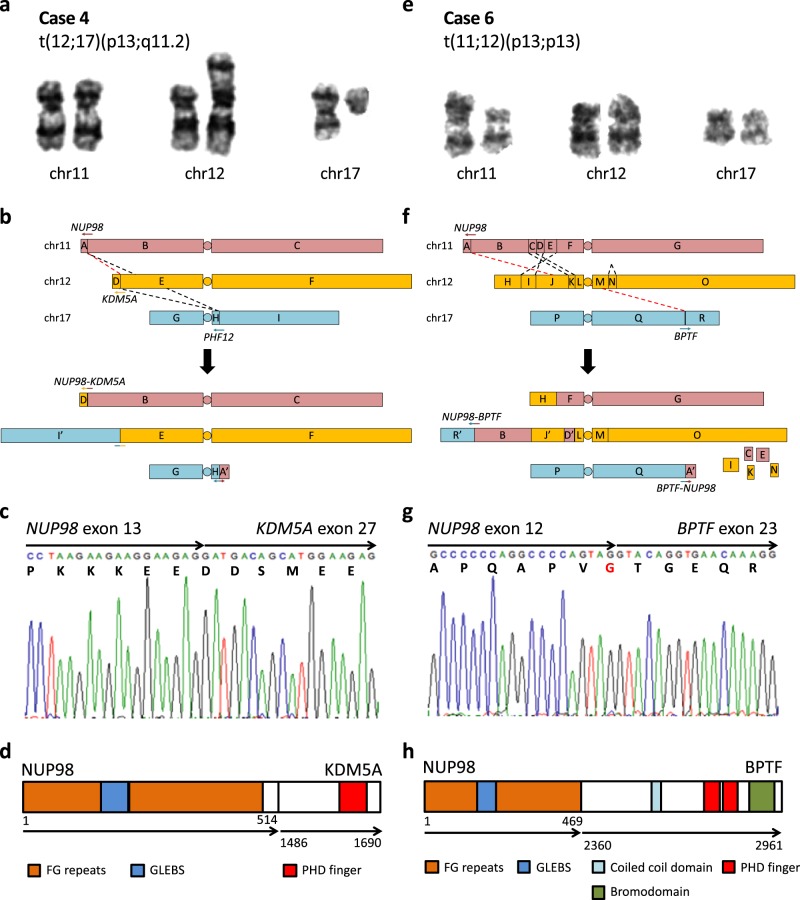
Cryptic *NUP98-KDM5A* and *NUP98-BPTF* fusion events in case 4 (left panels) and case 6 (right panels), respectively. **a**, **e** Partial karyograms of chromosomes 11, 12, and 17 and relevant cytogenetic findings. **b**, **f** Schematic representations of SVs in aforementioned chromosomes. Rearranged chromosomes, predicted from SVs and cytogenetics, are shown below. Arrows represent genes in 5′ to 3′ direction, alphabets represent genomic segments demarcated by case-specific breakpoints, apostrophes represent inverted segments, dashed lines represent SVs, and red dashed lines represent SVs leading to gene fusions. **c**, **g** Reverse transcription (RT)-PCR and Sanger sequencing of fusion transcripts. Arrows represent fused exons in 5′ to 3′ direction and letters below nucleotide codons represent corresponding amino acids. **d, h** Predicted fusion proteins and their domain structures adapted from UniProt. Arrows represent fused proteins and numbers represent amino acid positions

Notably, four out of nine leukemias in our cohort harbored gene fusion events that were not identified by cytogenetics: *ETV6-MECOM* (case 3), *NUP98-KDM5A* (case 4), *PICALM-MLLT10* (case 5), and *NUP98-BPTF* (case 6) (Fig. 1, Supplementary Fig. 2a, and Supplementary Table 4). The latter three created in-frame fusion transcripts, but *ETV6-MECOM*, similar to previously reported cases, created an out-of-frame fusion between intron 4 of *ETV6* (NM_001987) and intron 1 of *MECOM* (NM_001205194) that led to increased expression of *EVI1/MECOM* from an alternative translation start site in exon 3. All four cases were diagnosed with de novo AML and had adverse outcome despite receiving intensive induction therapy as per institutional protocol; cases 3, 5, and 6 were primary refractory and case 4 died during induction. These four fusions are known markers of poor prognosis, for which no targeted therapies currently exist [6–8]. While *ETV6-MECOM* and *PICALM-MLLT10* are not uncommon in AML, *NUP98* fusions are collectively found in only 2–4% of AML cases [6, 9]. To our knowledge, case 6 represents the third reported leukemia patient with a *NUP98-BPTF* fusion (Supplementary Table 5). The first report of *NUP98-BPTF* was in a young adult with T-cell acute lymphoblastic leukemia (ALL) [10] and the second was in an infant with acute megakaryoblastic leukemia [11]; both fusions were identified via RNA-seq. Clinical presentations of these three *NUP98-BPTF* cases are in line with the observation that *NUP98* fusions can occur in both myeloid neoplasms and T-cell ALL. Interestingly, cells from the first two cases were also characterized by complex karyotypes. Based on the shared presence of complex karyotype and the proximity of *NUP98* to the telomere, we speculate that the prevalence of *NUP98-BPTF* may be underestimated.

*NUP98* encodes a component of the nuclear pore complex and forms fusions with at least 31 different partner genes, many of which contain recurrent protein domains, such as homeodomain (HD) and plant homeodomain (PHD) [6, 9]. In particular, the PHD domain, which is a specific binder of trimethylated histone 3 lysine 4, is found in six of the partner genes including *KDM5A* and *BPTF* [9, 12] (Fig. 1d, h and Supplementary Fig. 3). PHD domains of both NUP98-KDM5A and wild-type BPTF are essential in the activation of *HOX* family genes, which is associated with stemness and poor prognosis [12–14]. NUP98-BPTF fusion in case 6 retains the C-terminal PHD domains of BPTF, so we predict that it can activate *HOX* genes, as previously investigated by Roussy et al. [11]. In addition, PICALM-MLLT10 in case 5 is another known activator of *HOX* [8], suggesting that *HOX* activation may be a common leukemogenic mechanism in the context of complex karyotype.

A later timepoint sample was available from case 5 whose disease progressed 10 months after diagnosis with marked increases in CD33 and CD117 antigens (Supplementary Table 6). WGS and RNA-seq were performed on this later sample to identify genomic changes that might be associated with the progression. Upon progression, a large deletion in 6q16.1-q22.31 was lost and a cryptic 4q12 deletion, resulting in the *FIP1L1*-*PDGFRA* fusion, was acquired (Supplementary Fig. 2b). This fusion constitutively activates PDGFRA and is a target of imatinib, a tyrosine kinase inhibitor [15]. In keeping with the growth factor independent state provided by the fusion transcript, the blast count rose from 41 × 10^9^/L at presentation to 464 × 10^9^/L at progression. This is to our knowledge the first report of *FIP1L1-PDGFRA*, an oncogenic driver, arising as a cooperating event during AML progression. It is possible that the progressed leukemia could have been controlled with imatinib, but we cannot predict the response of the diagnostic clone, which only carries *PICALM-MLLT10* and presumably is not driven by an activated receptor tyrosine kinase.

We next focused on the karyotypes and genomes of four cases with fusions to infer the reasons that made them cytogenetically invisible. In case 3, while sequencing detected a balanced translocation between 12p13.2 (*ETV6*) and 3q26.2 (*MECOM*) that was missed by cytogenetics, karyotyping identified three abnormalities, add(3)(q27), del(3)(q27), and add(12)(p13), near the fusion breakpoints. Due to the complexity of this case’s karyotype, which contains 31 abnormalities, it was probably not possible to discern the *ETV6-MECOM* rearrangement. For case 5, t(10;11)(p12.31;q14.2) translocation that creates the *PICALM-MLLT10* fusion was reported as t(10;11)(p1?2;q21), highlighting the limits of cytogenetic resolution in nonstimulated bone marrow cultures. For case 4, cytogenetics reported t(12;17)(p13;q11.2) but genomic analysis revealed a three-way translocation t(11;12;17)(p15.4;p13.3;q11.2) with *NUP98-KDM5A* fusion arising from the t(11;12) (Fig. 1a, b). The breakpoints for *NUP98* (11p11.5) and *KDM5A* (12p13.33) were cryptic because they are both terminal G-light material with very short rearranged segments (3.7 and 0.4 Mb, respectively) and below the resolution limit of G-banding [2]. For case 6, genomic investigation revealed a complex rearrangement pattern involving five interchromosomal translocations between chromosomes 11, 12, and 17 and a deletion in chromosome 12 (Fig. 1e, f). Collectively, they led to the loss of five chromosomal segments and the joining of *NUP98* at 11p15.4 to *BPTF* at 17q24.2. One of the five translocations joined *ETV6* at 12p13.2 to *LRRC4C* at 11p12 and could be ascribed to t(11;12)(p13;p13) from the karyotype, but it did not produce a functional, in-frame fusion transcript. Overall, three factors contributed to making a fusion event cytogenetically cryptic: the high number of cytogenetic abnormalities in a complex karyotype case, the proximity of a breakpoint to the telomere which tends to be G-light, and the complex rearrangement pattern, which conceals the fusion-causing translocation. An approach combining WGS and RNA-seq is advantageous in that it can bypass these factors in detecting gene fusions.

Our findings support the utility of integrated analysis of WGS and RNA-seq to identify genomic lesions of clinical importance that are undetected or incompletely revealed by cytogenetics. We provide further evidence that fusions resulting from the exchange of distal segments are difficult to ascertain by conventional cytogenetics. With continuing decrease in sequencing costs and increase in sequencing capacity, low-coverage WGS and RNA-seq are cost-effective methods to complement cytogenetics and provide a more complete picture of each leukemia. Furthermore, detecting gene fusions by sequencing can reveal a therapeutic target and/or provide markers for residual disease monitoring. The uncovering of known, and yet elusive, biomarkers in AML using genomics technologies argues for a more routine use of these established methods to help in understanding and accurately defining adverse-risk leukemias.

## Supplementary information

Supplementary Information
