# Supplementary material for: Cryptic genomic lesions in adverse-risk acute myeloid leukemia identified by integrated whole genome and transcriptome sequencing
Source: Leukemia. 2019 Aug 21;34(1):306–11. doi: 10.1038/s41375-019-0546-1 (PMC7214252; doi:10.1038/s41375-019-0546-1)
Supplement: Supplementary file 1 — Supplementary Information [file 41375_2019_546_MOESM1_ESM.pdf]

# SUPPLEMENTARY INFORMATION

# CRYPTIC GENOMIC LESIONS IN ADVERSE-RISK ACUTE MYELOID LEUKEMIA IDENTIFIED BY INTEGRATED WHOLE GENOME AND TRANSCRIPTOME SEQUENCING

Jaeseung C. Kim[1,2,3], Philip C. Zuzarte[2], Tracy Murphy[3], Michelle Chan-Seng-Yue[3,4], Andrew M. K. Brown[2], Paul M. Krzyzanowski[2], Adam C. Smith[5], Faiyaz Notta[1,3,4], Mark D. Minden[1,3], and John D. McPherson[1,2,6]

[1]Department of Medical Biophysics, Faculty of Medicine, University of Toronto, Toronto, ON, Canada;

[2]Genomics Technology Program, Ontario Institute for Cancer Research, Toronto, ON, Canada;

[3]Princess Margaret Cancer Centre, University Health Network, Toronto, ON, Canada;

[4]PanCuRx Translational Research Initiative, Ontario Institute for Cancer Research, Toronto, ON, Canada;

[5]Department of Laboratory Medicine and Pathobiology, University of Toronto and the Laboratory Medicine Program, University Health Network, Toronto, ON, Canada;

[6]Biochemistry and Molecular Medicine, University of California, Davis, Sacramento, CA, USA

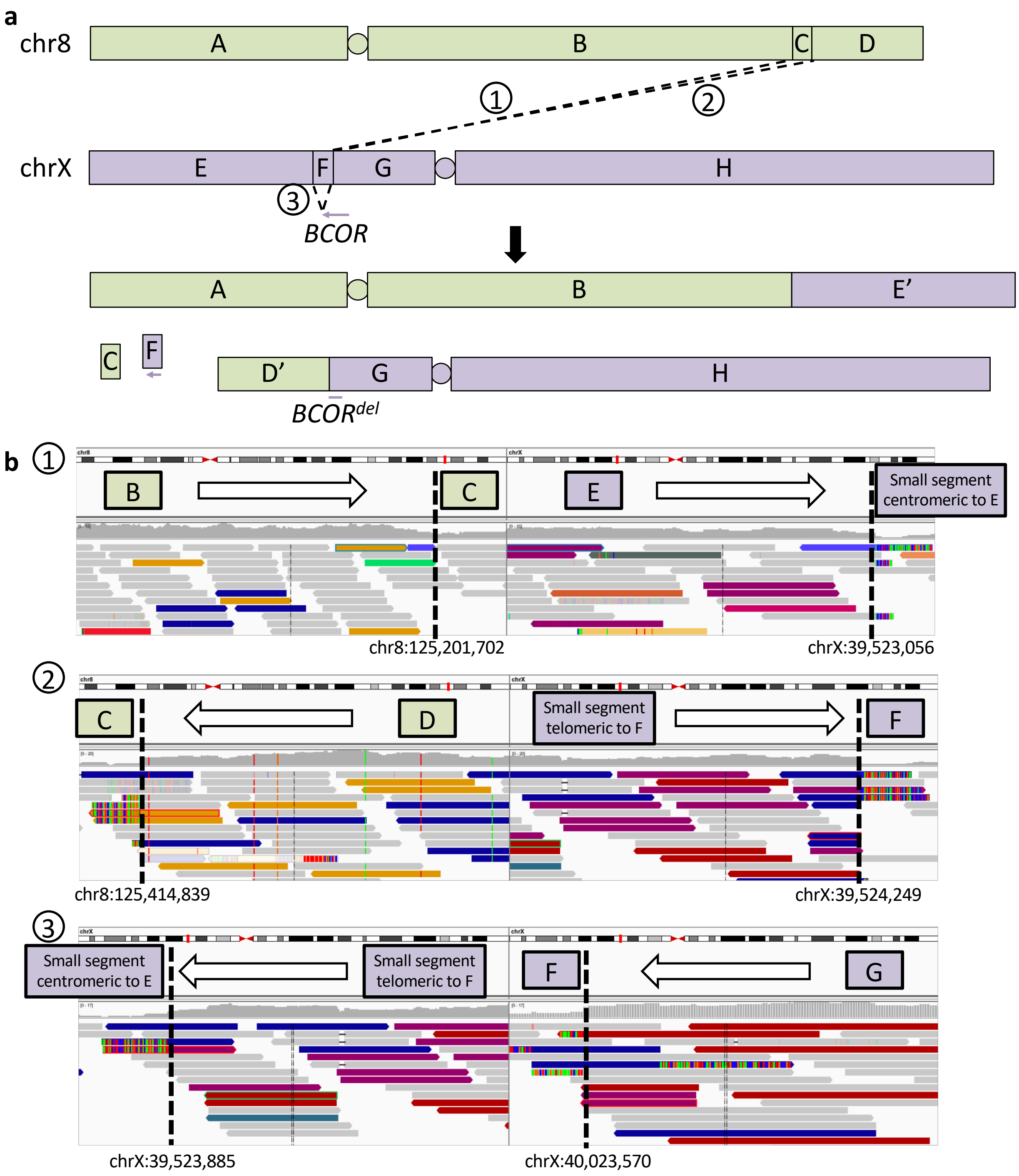


**Supplementary Figure 1.** Three SVs involved in t(X;8) and *BCOR* deletion in case 2. **a** Schematic representation of SVs as in Fig. 1 b, f. **b** Integrative Genomics Viewer (IGV) visualization of sequencing reads involved in each SVs. Boxes represent genomic segments, dashed lines represent breakpoints, and arrows represent directions of discordant Illumina paired-end reads. Orientations of rearranged genomic segments in **a** are inferred by re-orienting read pairs to point towards each other.

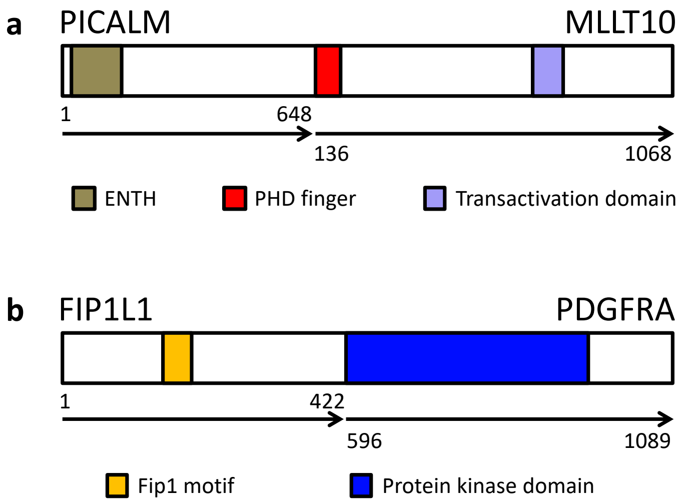


**Supplementary Figure 2.** Domain structures of **a** PICALM-MLLT10 and **b** FIP1L1-PDGFRA fusion proteins from case 5. See Fig. 1d, h for legends.

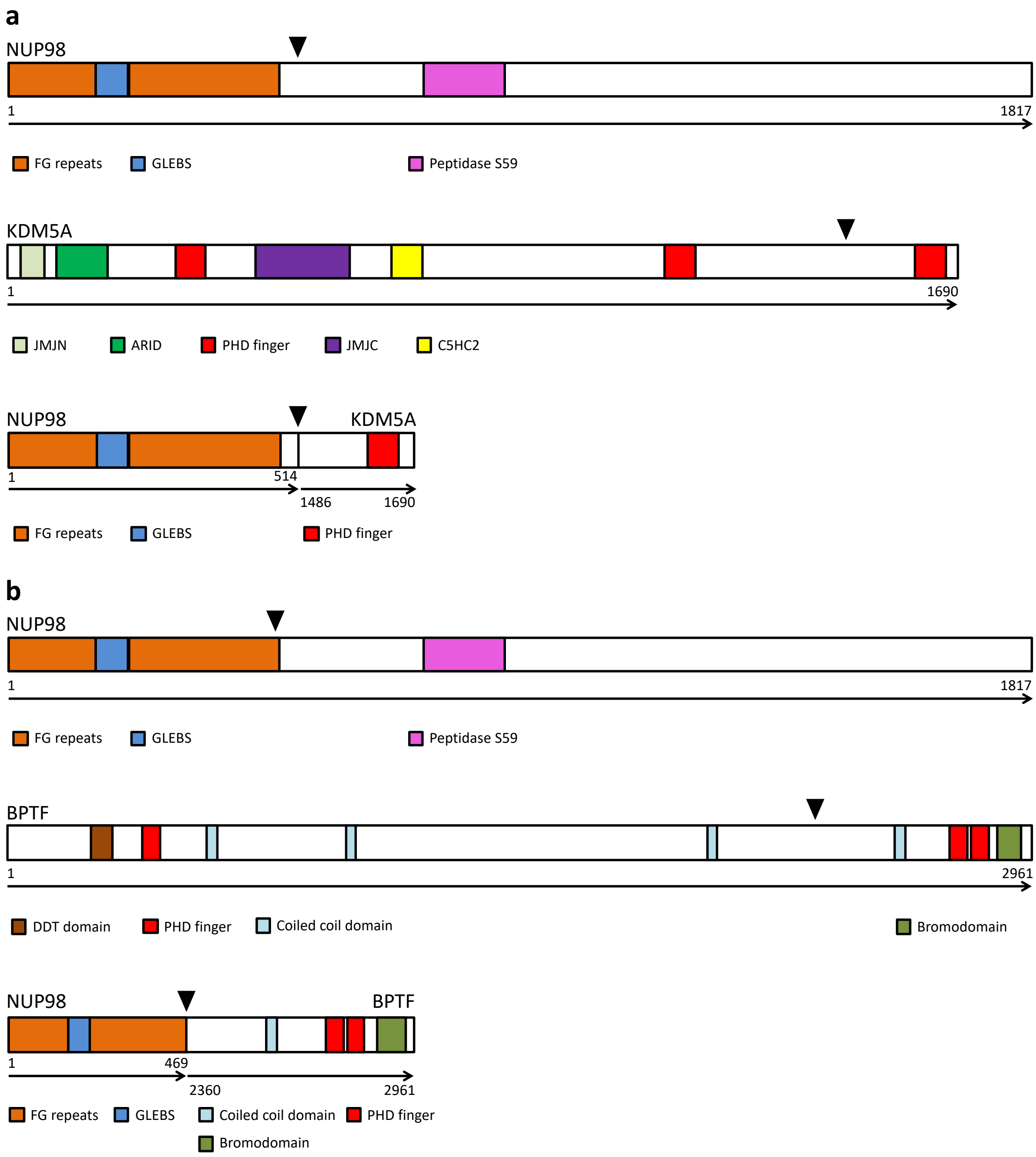


**Supplementary Figure 3.** Domain structures of proteins involved in **a** NUP98-KDM5A and **b** NUP98-BPTF fusions. Triangles represent fusion junctions. See Fig. 1d, h for legends.

**Supplementary Table 1.** Clinical data and additional summary of genomic findings from nine patients with adverse-risk AML

| Case | Gender | Age | UHN Biobank ID | Diagnosis | Induction therapy | Primary refractory (Yes/No) | WGS coverage | # of chromosomal gains/losses | | # of sub-chromosomal gains/losses | | # of translocations/ inversions | |
|---|---|---|---|---|---|---|---|---|---|---|---|---|---|
| | | | | | | | | Total | NGS concordant | Total | NGS concordant | Total | NGS concordant |
| 1 | M | 55 | 0187 | Secondary AML following MDS | 3+7 | No; died due to sepsis during induction | 15.5 | 4 | 4 | 0 | 0 | 1 | 1 |
| 2 | F | 54 | 0619 | AML (T/myeloid phenotype) | 3+7 | Yes | 10.5 | 1 | 1 | 0 | 0 | 1 | 1 |
| 3 | M | 64 | 5726 | AML | 3+7 + lovostatin | Yes | 14.9 | 7 | 2 | 20 | 6 | 4 | 1 |
| 4 | F | 42 | 8213 | AML | 3+7 | No; died due to ICH during induction | 10.1 | 3 | 3 | 0 | 0 | 3 | 2 |
| 5 | M | 33 | 8423 | AML | 3+7 | Yes | 9.6 | 0 | 0 | 2 | 1 | 2 | 2 |
| 6 | F | 20 | 8886 | AML | 3+7 | Yes | 11.6 | 1 | 1 | 1 | 1 | 1 | 1 |
| 7 | M | 46 | 080110 | APL-like | 3+7 | Yes | 11.2 | 6 | 3 | 0 | 0 | 2 | 2 |
| 8 | F | 64 | 522222 | AML | 3+7 | Yes | 6.5 | 6 | 1 | 4 | 2 | 4 | 3 |
| 9 | F | 70 | 130053 | AML | 3+7 | Yes | 11.9 | 0 | 0 | 0 | 0 | 1 | 1 |

| | Chromosomal gains/losses | Sub-chromosomal gains/losses | Translocations/ inversions |
|---|---|---|---|
| Concordance rate | 53.6% | 37.0% | 73.7% |

**Supplementary Table 2.** List of bioinformatics tools used in this study and their references

| Algorithm | Version | Purpose | Reference |
|---|---|---|---|
| BWA | 0.7.4 | Whole-genome sequencing alignment | Li H, Durbin R. Fast and accurate short read alignment with Burrows-Wheeler transform. Bioinformatics 2009; 25: 1754–1760. |
| CREST | 1.0 | Structural variant detection | Wang J, Mullighan CG, Easton J, Roberts S, Heatley SL, Ma J et al. CREST maps somatic structural variation in cancer genomes with base-pair resolution. Nat Methods 2011; 8: 652–654. |
| HMMcopy | 0.1.1 | Copy number analysis | Ha G, Roth A, Lai D, Bashashati A, Ding J, Goya R et al. Integrative analysis of genome-wide loss of heterozygosity and monoallelic expression at nucleotide resolution reveals disrupted pathways in triple-negative breast cancer. Genome Res 2012; 22: 1995–2007. |
| STAR | 2.5.2a | RNA-seq alignment | Dobin A, Davis CA, Schlesinger F, Drenkow J, Zaleski C, Jha S et al. STAR: ultrafast universal RNA-seq aligner. Bioinformatics 2013; 29: 15–21. |
| STAR-Fusion | 1.0.0 | Fusion transcript detection | Haas B, Dobin A, Stransky N, Li B, Yang X, Tickle T et al. STAR-Fusion: Fast and Accurate Fusion Transcript Detection from RNA-Seq. bioRxiv (Preprint) 2017. doi:10.1101/120295. |
| GATK | 3.6.0 | SNV and indel calling | Poplin R, Ruano-Rubio V, DePristo MA, Fennell TJ, Carneiro MO, Van der Auwera GA et al. Scaling accurate genetic variant discovery to tens of thousands of samples. bioRxiv (Preprint) 2018. doi:10.1101/201178. |
| IGV | 2.3.88 | Visualization of sequencing data | Thorvaldsdottir H, Robinson JT, Mesirov JP. Integrative Genomics Viewer (IGV): high-performance genomics data visualization and exploration. Briefings in Bioinformatics 2013; 14: 178–192. |

**Supplementary Table 3.** Individual cytogenetic abnormalities and concordant NGS findings

| Case | Karyotype (NGS concordant abnormalities listed underneath) | NGS findings | | |
|---|---|---|---|---|
| | | Copy number gain | Copy number loss | Chromosomal rearrangement |
| 1 | 51, XY, **+Y**, **+der(1;7)(q10;p10)**, **+6**, **+8**, **+10** [10] | | | |
| | **+Y** | **Y** | | |
| | **+der(1;7)(q10;p10)** | **1q, 7p** | | |
| | **+6** | **6** | | |
| | **+8** | **8** | | |
| | **+10** | **10** | | |
| 2 | 45, XX, **-7** [17] / 45, **X**, **t(X;8)(p21.2;q24.1)**, **-7** [2] / 46, XX[1] | | | |
| | **-7** | | **7** | |
| | **t(X;8)(p21.2;q24.1)** | | *BCOR* | **t(X;8)(p11.4;q24.13)** linked to *BCOR* deletion |
| 3 | 43~45, **X**, **-Y**, t(1;5)(q21;p13), t(1;6)(q21;p25), -2, **add(2)(p21)**, **add(3)(q27)**, **del(3)(q27)**, add(4)(p16), del(4)(q33), del(5)(q31), add(6)(p25), del(6)(q21), -7, add(7)(q36), **del(7)(q22)**, -8, **add(8)(q24.3)**, del(8)(?q23), +del(8)(q22), add(9)(p22), add(9)(q34), add(11)(p15), **add(12)(p13)**, -16, add(16)(p13.3), **-17**, **der(17)t(11;17)(q13;p13)**, add(18)(q23), der(19)t(11;19)(q13;q13.3), -21, add(22)(p11.2), +4mar [cp20] | | | |
| | **-Y** | | **Yq11.221-q12** | |
| | **add(2)(p21)** | **2p21-p11.2** (*BCL11A*) | | |
| | **add(3)(q27)** | **3q26.32-q29** | | |
| | **del(3)(q27)** | | | chr**2**/**3**/**8**/**11**/**12**/16/ **17**/19/X rearrangements |
| | **del(7)(q22)** | | **7q21.3-q36.2** | |
| | **add(8)(q24.3)** | **8q12.1-q23.1** (*RUNX1T1*) | | |
| | **add(12)(p13)** | **12p13.33-p13.2** | | |
| | **-17** | | **17p13.3-p12** (*TP53*), **17q11.2-q24.3** (*NF1*) | |
| | **der(17)t(11;17)(q13;p13)** | **11q13.4-q25** (*ETS1*) | | chr**2**/**3**/**8**/**11**/**12**/16/ **17**/19/X rearrangements |
| 4 | 48-49, XX, **+6**, **+8**, **+9**, **t(12;17)(p13;q11.2)**, i(17)(q10), **inv(18)(q11.2q21)** [cp20] | | | |
| | **+6** | **6** | | |
| | **+8** | **8** | | |
| | **+9** | **9** | | |
| | **t(12;17)(p13;q11.2)** | | | t(11;**12**;**17**)(p15.4;**p13.3;** **q11.2**) linked to gene fusion |
| | **inv(18)(q11.2q21)** | | **18q21.2** (*SMAD4*) | |
| 5 | 46, XY, **del(6)(q21q23)**, **t(10;11)(p1?2;q21)**, **inv(12)(q15q24.1)**, del(15)(q2?4) [cp19] / 46, XY [1] | | | |
| | **del(6)(q21q23)** | | **6q16.1-q22.31** (*FOXO3*) | |
| | **t(10;11)(p1?2;q21)** | | | **t(10;11)**(p12.3;q14.2) linked to gene fusion |
| | **inv(12)(q15q24.1)** | | | **chr12 rearrangements** linked to *SH2B3* and *TBX3* deletions |
| 6 | 46, XX, **del(5)(q31)**, **t(11;12)(p13;p13)** [4] / 45, **idem**, **-13** [5] / 46, XX [1] | | | |
| | **del(5)(q31)** | | **5q22.2q32** (*APC*) | |
| | **t(11;12)(p13;p13)** | | | chr**11**/**12**/17 rearrangements linked to CNVs and gene fusion (Figure 1) |
| | **-13** | | **13** | |

| Case | Karyotype (NGS concordant abnormalities listed underneath) | NGS findings | | |
|---|---|---|---|---|
| | | Copy number gain | Copy number loss | Chromosomal rearrangement |
| 7 | 43~48, XY, +?Y, +?Y, **der(5)t(5;17)(q1?3;q11.2)**, **-7**, **+8**, **-12**, -13, **t(16;18)(q24;q23)**, +1~2mar [cp10] | | | |
| | **der(5)t(5;17)(q1?3;q11.2)** | | | chr2/**5**/**12**/13/**16**/**17**/**18** rearrangements |
| | **-7** | | **7** | |
| | **+8** | **8** | | |
| | **-12** | | **12p13.2-p12.1** (*ETV6*), **12p12.1-p11.21**, **12q12-q24.13** | chr2/**5**/**12**/13/**16**/**17**/**18** rearrangements |
| | **t(16;18)(q24;q23)** | | | chr2/**5**/**12**/13/**16**/**17**/**18** rearrangements |
| 8 | 42-45, X, der(X)t(X;3)(q28;q21), **der(1)t(1;17)(p35;q21)**, **-3**, -5, **add(8)(q13)**, -9, add(11)(q13), add(12)(q24), **i(13)(q10)**, **del(16)(q23)**, -17, -18, -19, **+idic(22)(p12)**, +2r, +mar1 [cp9] / 46, XX [1] | | | |
| | **der(1)t(1;17)(p35;q21)** | | | chr**1**/5/**8**/9/11/12/**16**/**17**/19 rearrangements |
| | **-3** | | **3** | |
| | **add(8)(q13)** | **8q** (*MYC*) | | chr**1**/5/**8**/9/11/12/**16**/**17**/19 rearrangements |
| | **i(13)(q10)** | **13** | | |
| | **del(16)(q23)** | | **16q21-q24.3** (*CTCF*) | chr**1**/5/**8**/9/11/12/**16**/**17**/19 rearrangements |
| | **+idic(22)(p12)** | **22** | | |
| 9 | 46, XX, **t(1;14)(q21;q11.2)** [20] | | | |
| | **t(1;14)(q21;q11.2)** | | | **t(1;14)(q23.2;q12)** linked to truncation of *ATP1A2* |

**Supplementary Table 4.** Cross-analysis of WGS and RNA-seq findings for gene fusion detection

| Case | WGS | | | | | | | | | RNA-seq | | | | | | | | | | Description | Driver gene fusion |
|---|---|---|---|---|---|---|---|---|---|---|---|---|---|---|---|---|---|---|---|---|---|
| | Left chr | Left pos | Left strand | Right chr | Right pos | Right strand | Left gene | Right gene | Gene orientation | Left chr | Left pos | Left strand | Right chr | Right pos | Right strand | Left gene | Right gene | Junction read # | Spanning fragment # | | |
| 2 | chr3 | 17738725 | - | chr15 | 34634338 | + | TBC1D5 | NOP10 | Opposite | chr3 | 17783973 | - | chr15 | 34640170 | + | TBC1D5 | NUTM1 | 28 | 109 | Out-of-frame; 5′ UTR of *TBC1D5* | |
| | chrX | 40023570 | - | chrX | 39523885 | + | BCOR | | | | | | | | | | | | | #3 in Supp. Fig. 1; linked to *BCOR-RNF139* below | |
| | chrX | 39524250 | + | chr8 | 125414839 | + | | | | | | | | | | | | | | #2 in Supp. Fig. 1; linked to *BCOR-RNF139* below | |
| | | | | | | | | | | chrX | 40036260 | - | chr8 | 125498072 | + | BCOR | RNF139 | 4 | 2 | Out-of-frame; 5′ UTR of *BCOR* | |
| 3 | chr20 | 45972885 | - | chr9 | 132665158 | - | ZMYND8 | FNBP1 | Same | chr20 | 45976600 | - | chr9 | 132662826 | - | ZMYND8 | FNBP1 | 88 | 210 | Out-of-frame | |
| | chr12 | 12012826 | + | chr3 | 168875442 | - | ETV6 | MECOM | Same | chr12 | 12006495 | + | chr3 | 168861620 | - | ETV6 | MECOM | 18 | 75 | Out-of-frame | Increased expression of *MECOM* from an alternative translation start site |
| | chr5 | 66263319 | + | chr1 | 235611175 | + | MAST4 | TBCE | Same | | | | | | | | | | | Out-of-frame | |
| | chr16 | 57065493 | + | chr19 | 2275316 | - | NLRC5 | C19orf35 | Same | | | | | | | | | | | 3′ UTR of *C19orf35* | |
| | chr17 | 35936207 | + | chr17 | 30854871 | + | SYNRG | MYO1D | Same | | | | | | | | | | | Out-of-frame | |
| | chr7 | 138769597 | - | chr7 | 139432554 | - | HIPK2 | ZC3HAV1 | Same | chr7 | 138774370 | - | chr7 | 139416814 | - | ZC3HAV1 | HIPK2 | 26 | 48 | Out-of-frame | |
| | chr17 | 56794315 | - | chr17 | 57090343 | + | RAD51C | TRIM37 | Same | | | | | | | | | | | Out-of-frame; *TRIM37* is massively rearranged | |
| | chr17 | 57018384 | + | chr17 | 57102178 | - | PPM1E | TRIM37 | Same | | | | | | | | | | | Out-of-frame; *TRIM37* is massively rearranged | |
| | chr7 | 138565338 | + | chr4 | 151196527 | + | KIAA1549 | LRBA | Same | | | | | | | | | | | In-frame; *LRBA* is massively rearranged; no fusion transcript | |
| | | | | | | | | | | chr4 | 151814204 | - | chr7 | 138768778 | - | LRBA | ZC3HAV1 | 20 | 54 | Out-of-frame | |
| | | | | | | | | | | chr7 | 139313699 | - | chr4 | 151719294 | - | HIPK2 | LRBA | 18 | 62 | Out-of-frame | |
| | | | | | | | | | | chr7 | 139030367 | + | chr4 | 151186964 | - | LUC7L2 | LRBA | 7 | 43 | Out-of-frame | |
| | chr16 | 80782 | - | chr12 | 10531157 | - | IL9RP3 | KLRK1 | Same | chr16 | 81799 | - | chr12 | 10530834 | - | IL9RP3 | KLRK1 | 3 | 2 | In-frame; *IL9RP3* is a pseudogene | |
| 4 | chr11 | 3753668 | - | chr12 | 404641 | + | NUP98 | KDM5A | Same | chr11 | 3756421 | - | chr12 | 402335 | - | NUP98 | KDM5A | 44 | 78 | In-frame | Yes |
| 5 | chr11 | 85680853 | - | chr10 | 21888265 | + | PICALM | MLLT10 | Same | chr11 | 85685751 | - | chr10 | 21901277 | + | PICALM | MLLT10 | 62 | 51 | In-frame | Yes |
| | chr10 | 21888246 | + | chr11 | 85680578 | - | MLLT10 | PICALM | Same | chr10 | 21884369 | + | chr11 | 85670103 | - | MLLT10 | PICALM | 23 | 99 | In-frame | |
| 6 | chr11 | 3759564 | - | chr17 | 65941452 | + | NUP98 | BPTF | Same | chr11 | 3765739 | - | chr17 | 65941525 | + | NUP98 | BPTF | 28 | 22 | In-frame | Yes |
| | chr17 | 65941478 | + | chr11 | 3758816 | - | BPTF | NUP98 | Same | chr17 | 65940488 | + | chr11 | 3756554 | - | BPTF | NUP98 | 7 | 46 | In-frame | |
| | chr6 | 108833664 | + | chr6 | 108890015 | + | LACE1 | FOXO3 | Same | | | | | | | | | | | In-frame; no fusion transcript; low expression of *LACE1* | |
| 7 | chr13 | 21382331 | - | chr8 | 41470742 | + | XPO4 | AGPAT6 | Same | | | | | | | | | | | Out-of-frame | |
| | chr16 | 68808494 | + | chr12 | 111922848 | - | CDH1 | ATXN2 | Same | | | | | | | | | | | In-frame; no fusion transcript; no expression of *CDH1* | |
| | chr17 | 36953437 | + | chr17 | 36710058 | + | PIP4K2B | SRCIN1 | Same | | | | | | | | | | | Out-of-frame | |
| | chr1 | 22453096 | + | chr1 | 28159637 | - | WNT4 | PPP1R8 | Same | | | | | | | | | | | Out-of-frame | |
| | chr2 | 86740965 | - | chr18 | 75954665 | + | CHMP3 | | | | | | | | | | | | | Linked to *CHMP3-CDH3* below | |
| | chr18 | 75966176 | + | chr16 | 68756154 | + | | CDH3 | | | | | | | | | | | | Linked to *CHMP3-CDH3* below | |
| | | | | | | | | | | chr2 | 86756341 | - | chr16 | 68756328 | + | CHMP3 | CDH3 | 51 | 114 | In-frame; putative alternative transcript of *CDH3* | |
| | chr7 | 157134847 | + | chr7 | 71504707 | - | CALN1 | DNAJB6 | Same | chr7 | 157129838 | + | chr7 | 71488754 | - | DNAJB6 | CALN1 | 12 | 42 | Inversion; out-of-frame; 5′ UTR of *DNAJB6* | |
| 8 | chr9 | 115804777 | + | chr1 | 19501815 | + | ZFP37 | UBR4 | Same | | | | | | | | | | | 3′ UTR of *ZFP37* | |
| | chr8 | 40573882 | - | chr12 | 27101439 | + | ZMAT4 | FGFR1OP2 | Same | | | | | | | | | | | Out-of-frame; 5′ UTR of *FGFR1OP2* | |
| | chr11 | 120815777 | + | chr11 | 121009286 | + | GRIK4 | TECTA | Same | | | | | | | | | | | In-frame; no fusion transcript; no expression of both genes | |
| | chr16 | 89500468 | - | chr17 | 28688888 | + | ANKRD11 | | | chr16 | 89556653 | - | chr17 | 28712007 | + | ANKRD11 | CPD | 3 | 3 | Out-of-frame; 5′ UTR of *ANKRD11* | |

**Supplementary Table 5.** Literature review of three cases with *NUP98-BPTF* fusion

| Case | 1 | 2 | 3 |
|---|---|---|---|
| Reference | Yasuda *et al*., 2016 | Roussy *et al*., 2018 | This study (#6) |
| Sex | Female | Male | Female |
| Age | 19 years | 8 months | 20 years |
| Diagnosis | T-cell acute lymphoblastic leukemia | Acute megakaryoblastic leukemia | Acute myeloid leukemia |
| Karyotype | 86~89, XXXX, +X, add(1)(p36), -4, -5, -6, ?add(6)(q21), -7, -8, -9, -10, -11, der(11)add(11)(p11) add(11)(q23), -13, -14, -15, -16, -17, -18, -18, -20, -21, +11, ~15mar, inc [cp5] / 46, XX [14] | 49, XY, +6, +7, t(11;17)(p15;q23), +19 | 46, XX, del(5)(q31), t(11;12)(p13;p13) [4] / 45, idem, -13 [5] / 46, XX [1] |
| *NUP98* exons | Not available | 1-11 | 1-12 |
| *BPTF* exons | Not available | 26-30* | 23-30* |

*NUP98* exons are derived from Refseq transcript NM_016320, and *BPTF* exons are derived from Refseq transcript NM_004459. *An additional 174-bp exon is included between exons 26 and 27 at chr17:65,959,449-65,959,622 (ref. 11).

**Supplementary Table 6.** Immunophenotype of case 5 at diagnosis and progression

| | Diagnosis | Progression |
|---|---|---|
| Myelomonocytic associated markers | | |
| CD11b | 0.32 | 0.42 |
| CD13 | 0.57 | 0.45 |
| CD15 | 0.02 | 0.01 |
| CD33 | 0.08 | 0.45 |
| CD34 | 0.38 | 0.51 |
| CD56 | <0.01 | 0.02 |
| CD65 | 0.03 | 0.01 |
| CD117 | 0.01 | 0.30 |
| HLA-DR | 0.92 | 0.91 |
| CD33+CD34+ | 0.06 | 0.40 |
| Lymphoid markers | | |
| CD2 | 0.04 | 0.03 |
| CD3 | 0.01 | <0.01 |
| CD4+CD3+ | <0.01 | NA |
| CD4 | NA | 0.03 |
| CD5 | 0.05 | NA |
| CD7 | >0.99 | 0.98 |
| CD8+CD3+ | <0.01 | NA |
| CD10 | 0.01 | <0.01 |
| CD19 | 0.06 | 0.17 |
| Intracellular markers | | |
| TdT | 0.01 | 0.01 |
| MPO | <0.01 | <0.01 |
| CyCD3 | 0.01 | 0.02 |
| CyCD79a | 0.61 | 0.43 |
| CyCD22 | 0.02 | NA |

Blasts are gated on CD45 versus side scatter. Blast region contains ~86% of gated cells at diagnosis and ~55% at relapse.
